# Principles of intracellular bacterial pathogen spread from cell to cell

**DOI:** 10.1371/journal.ppat.1007380

**Published:** 2018-12-13

**Authors:** Erin Weddle, Hervé Agaisse

**Affiliations:** Department of Microbiology, Immunology, and Cancer Biology, University of Virginia, Charlottesville, Virginia, United States of America; Duke University School of Medicine, UNITED STATES

A subset of intracellular pathogens, including *Listeria monocytogenes*, *Shigella flexneri*, *Rickettsia* spp., and *Burkholderia* spp. disseminate within nonphagocytic cells, such as epithelial and endothelial cells, through a process referred to as cell-to-cell spread [1]. These pathogens utilize the host cell actin cytoskeleton to move in the cytosol of infected cells and project into adjacent cells through formation of membrane protrusions. The formed protrusions resolve into vacuoles from which the pathogen escapes, thereby gaining access to the cytosol of adjacent cells (Fig 1). Here, we present the general principles and summarize the underlying mechanisms supporting this bacterial dissemination process.

**Fig 1:**
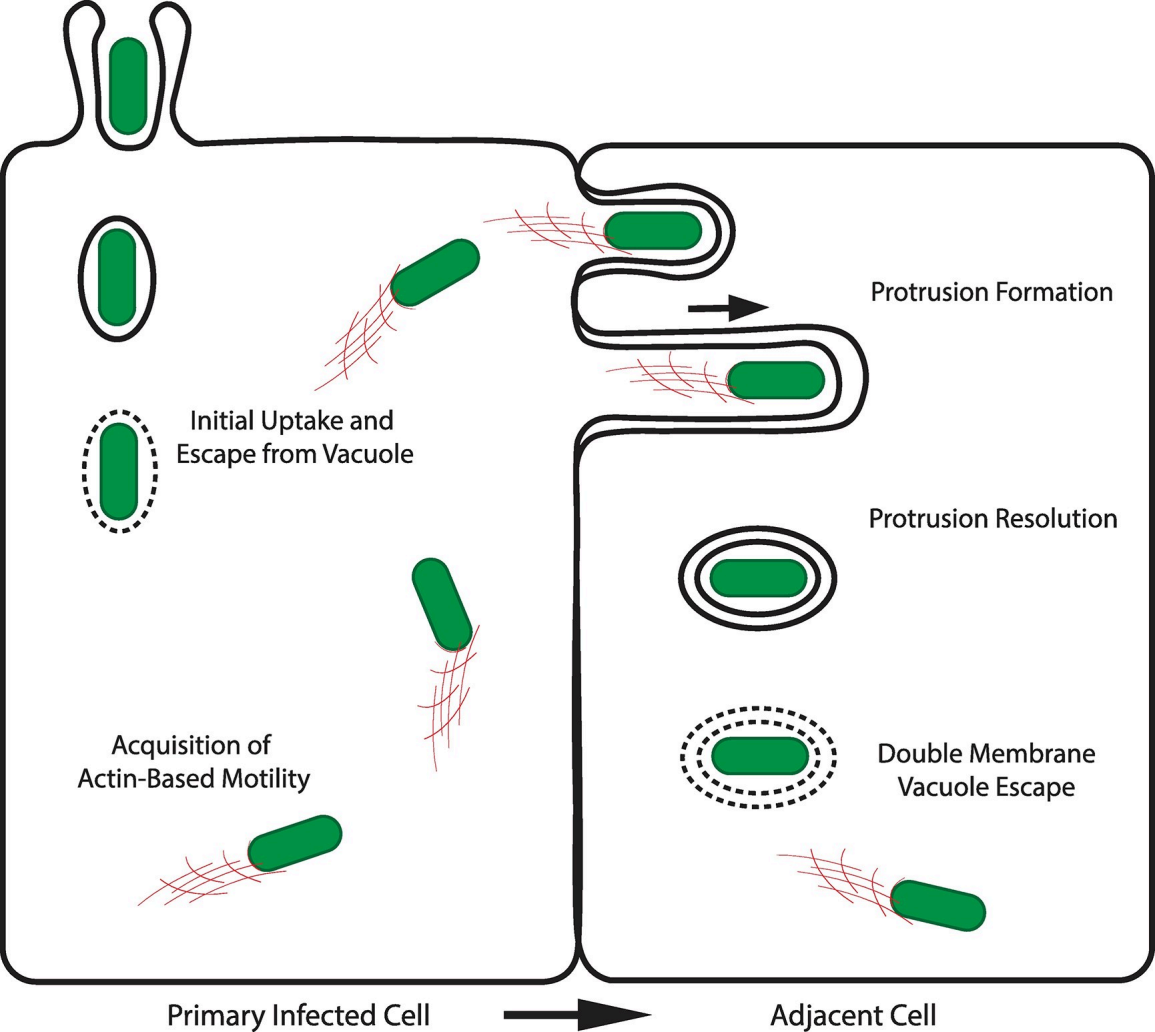
Intracellular bacterial spread from cell to cell. Steps supporting the intracellular dissemination of *Listeria monocytogenes* [46]. Important variations in this process during *Shigella flexneri*, *Rickettsia* spp., and *Burkholderia* spp. dissemination are discussed in this review.

## Step 1: Gaining access to the actin assembly machinery

Pathogenic bacteria gain access to the host cell actin assembly machinery through bacterial engulfment in membrane-bound compartments termed primary vacuoles from which the bacteria escape through secretion of bacterial factors that challenge the integrity of the vacuole membrane [2]. This invasion and vacuole escape process is a critical first step that grants the pathogens access to the cytosolic actin assembly machinery (Fig 1).

*L*. *monocytogenes* primary vacuole escape in human epithelial cells is facilitated by the production of the pore-forming toxin Listeriolysin O (LLO) [3] and phospholipases C (PlcA and PlcB) [4].

*S*. *flexneri* vacuole escape depends on the type-three secretion system (T3SS). Similar to *L*. *monocytogenes* LLO, the T3SS translocases, primarily IpaC, contribute to vacuole escape by forming pores in the vacuole membrane [5,6]. The host factor Rab11 has also been shown to contribute to *S*. *flexneri* vacuole escape [7]. The recruitment of Rab11 relies on the *S*. *flexneri* T3SS effector protein IpgD [7], suggesting that, in addition to the translocases, T3SS effector proteins are also involved in primary vacuole escape. *Rickettsia* spp. produce phospholipases that are implicated in vacuole escape; however, this mechanism is still unclear [8–11]. *Burkholderia* spp. escape vacuoles through the activity of the T3SS [12].

## Step 2: Acquisition of actin-based motility

Once in the cytosol, intracellular bacteria spread from cell to cell by first acquiring actin-based motility (ABM). In uninfected cells, actin polymerization relies on actin nucleators and their cognate regulators [13]. In infected cells, bacteria display ABM by hijacking host cell actin nucleators or by expressing bacterial actin nucleators [14]. These factors are localized at the bacterial pole on the bacterial surface, resulting in polar actin polymerization that propels the bacteria throughout the cytosol (Fig 1). Pathogens have evolved bacterial factors that mimic the activity of all types of host cell actin nucleators and cognate regulators known to date.

In *S*. *flexneri* and *L*. *monocytogenes*, the actin-related protein ARP2/3 complex—a critical host cell actin nucleator—is recruited to the bacterial pole. *S*. *flexneri* secretes a bacterial autotransporter protein IcsA (also known as VirG), whose activity recruits the ARP2/3 nucleation-promoting factor Wiskott−Aldrich Syndrome protein (N-WASP), and consequently ARP2/3, to the bacterial pole [15,16].

By contrast, *L*. *monocytogenes* expresses a bacterial factor, ActA [17], that recruits ARP2/3 at the bacterial pole [18] through structural and regulatory mimicry of N-WASP [19–21] and activates the ARP2/3 complex directly.

*Rickettsia* spp. encode RickA, an N-WASP mimic that recruits ARP2/3 to the bacterial surface and induces ABM [22,23]. In addition, *R*. *rickettsii* produce Sca2, which is required for cell-to-cell spread and resembles actin nucleators of the formin family [24]. While RickA leads to ARP2/3-mediated nucleation of branched actin filaments, Sca2 catalyzes the processive nucleation at the barbed end, producing networks of long, bundled actin filaments [25]. RickA is implicated in ABM early after invasion, whereas Sca2 is proposed to be the primary nucleator later in infection [26].

Similarly to *Listeria* and *Rickettsia*, *B*. *thailandensis* BimA acts as a nucleator that activates Arp2/3. However, orthologs of BimA from pathogenic counterparts *B*. *pseudomallei* and *B*. *mallei* mimic actin nucleators of the host Ena/VASP family [27]. Similar to VASP, BimA oligomerizes and binds multiple filaments to increase their elongation rate by outcompeting capping proteins [27].

## Step 3: Membrane protrusion formation

Cytosolic ABM allows intracellular pathogens to reach the plasma membrane at sites of cell−cell contacts, where they form membrane protrusions that project into adjacent cells (Fig 1). This process differs from ABM in the cytosol because (i) it requires countering tension at the plasma membrane and (ii) it occurs in a membrane-bound compartment that, as opposed to the cytosol, displays finite amounts of actin network components [28].

## Step 3a: Reducing tension at cell−cell contacts

*L*. *monocytogenes* releases Tuba/N-WASP-mediated tension at cell−cell contacts by secreting Internalin C (InlC), which binds Tuba, thereby displacing N-WASP [29]. The *R*. *parkeri* effector protein Sca4 has been proposed to release tension by interfering with vinculin−α-catenin interactions, potentially creating unequal actomyosin tension at cell junctions and promoting bacterial spread. This mechanism has been shown to contribute to protrusion resolution [30]. How *S*. *flexneri* and *Burkholderia* spp. overcome membrane tension is unknown.

## Step 3b: Protrusion elongation

In addition to the ARP2/3-dependent actin assembly machinery required for *L*. *monocytogenes* cytosolic ABM, membrane protrusion formation relies on the *AIP1/CFL1*-dependent disassembly machinery [28]. The disassembly of the distal actin network in membrane protrusions fuels the continuous actin assembly at the bacterial pole, a process termed local actin network recycling. Local recycling in a membrane-bound compartment is critical for efficient protrusion elongation [28]. Efficient membrane protrusion formation also requires host ERM family proteins [31] and formins [32], whose functions in protrusion are unknown.

It is presumed that, similar to cytosolic ABM, IcsA and N-WASP/Arp2-3 are responsible for actin polymerization in *S*. *flexneri* protrusions. In addition to ARP2/3, the host formins mDia1/2 localize to protrusions and are required for their proper formation [33]. Myosin-X also localizes to protrusions and was proposed to facilitate protrusion formation by bridging actin filaments and the plasma membrane [34].

Although *Rickettsia* spp. ABM relies on the actin cytoskeleton, it was recently observed that *R*. *parkeri* protrusions uniquely lack actin tails, suggesting that the *R*. *parkeri* protrusion formation may not rely on the forces generated by actin assembly [30]. This potentially actin-independent mechanism of protrusion formation remains to be elucidated.

Actin-containing membrane protrusions are formed during *B*. *pseudomallei* and *B*. *thailandensis* infection [12,35], but their exact contribution to the dissemination process remains unclear. It has been suggested that cell−cell fusion may support *Burkholderia* dissemination [12,35]. However, the potential contribution of membrane protrusions in the fusion process remains to be determined.

## Step 4: Resolution of protrusions into vacuoles in adjacent cells

During bacterial spread from cell to cell, membrane protrusions resolve into double membrane vacuoles (DMVs), whose inner and outer membranes are contributed by the primary infected cell and the adjacent cell, respectively (Fig 1). The formation of DMVs is a multistep process that requires the disassembly of the actin network (when involved) and the scission of the inner and outer membranes. Although the mechanisms supporting the scission of protrusion membranes remain poorly understood, the bacterial and cellular factors supporting the remodeling of the actin network in protrusions have been recently uncovered.

In *L*. *monocytogenes*, the host *AIP1/CFL1*-dependent disassembly machinery is critical not only for the formation but also for the resolution of protrusions [28]. It was proposed that local actin network recycling in protrusions allows for the generation of membrane tension through efficient actin polymerization at the bacterial pole, as well as exhaustion of the actin network in the distal part of protrusions, where membrane scission occurs [28]. The bacterial metallo-protease Mpl has been suggested to facilitate the resolution process through maturation of the bacterial nucleation-promoting factor ActA, although the exact role of ActA processing remains unknown [36]. An additional mechanism of protrusion resolution involving LLO, phosphatidylserine, and receptor T-cell immunoglobulin and mucin-domain containing protein 4 (TIM-4) has been described in macrophages [37]; however, the implication of this mechanism in *L*. *monocytogenes* spread in epithelial cells is unclear.

In *S*. *flexneri*, protrusions are resolved in a two-step process. The collapse of the protrusion neck, presumably due to the disassembly of the actin cytoskeleton network, results in the formation of intermediate structures termed vacuole-like protrusions (VLPs) [38]. Formation of VLPs requires several cellular signaling events, including tyrosine kinase and phosphoinositide signaling [38,39]. The subsequent severing of the VLP membrane tether leads to vacuole formation. On the bacterial side, tyrosine kinase and phosphoinositide signaling-dependent resolution of protrusions requires the integrity of the bacterial T3SS [40], but the T3SS effector proteins potentially involved have yet to be elucidated.

As mentioned above, the *Rickettsia* effector protein Sca4 contributes to *Rickettsia* spp. protrusion resolution [41]. Although *Burkholderia* spp. have been observed in protrusions, they have not been observed in DMVs [12]; therefore, the mechanism of protrusion resolution is unknown.

## Step 5: DMV escape

In order to resume ABM in the cytosol of adjacent cells, spreading bacterial pathogens must escape from the DMVs formed as a result of protrusion resolution. In contrast with primary vacuole escape, DMV escape is a complex process that requires the destabilization of two membranes (Fig 1).

*L*. *monocytogenes* accomplishes DMV escape by using pore-forming toxins and enzymes that challenge the integrity of the vacuole membranes [4]. Similar to primary vacuole escape, LLO and Plcs play seemingly complementary roles in DMV escape in human epithelial cells [42].

Because *S*. *flexneri* mutants lacking functional T3SS are trapped in DMVs, it was proposed that, similar to primary vacuole escape, the T3SS translocases may mediate DMV escape through pore formation [43]. In addition, the T3SS effector protein IcsB has recently been shown to be specifically required for effective DMV escape [44]. IcsB is an 18-carbon fatty acyltransferase that modifies several membrane-associated host proteins [45], although its exact function in DMV escape remains to be determined.

The mechanism supporting *Rickettsia* spp. DMV escape is poorly understood, and whether *Burkholderia* spp. forms DMVs altogether is unknown.

**Fig 2:**
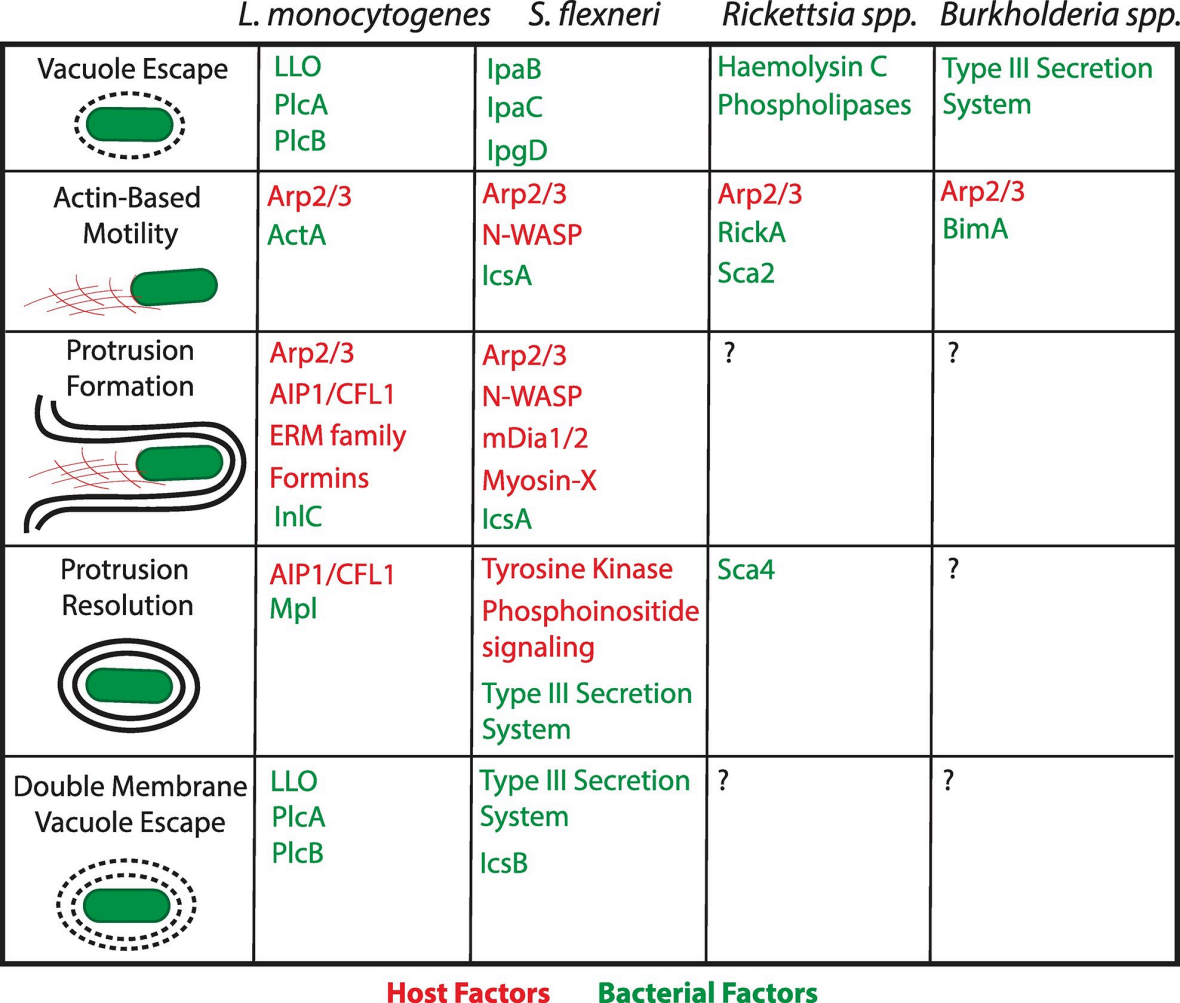
Host and bacterial factors that facilitate cell-to-cell spread. For each step of spread, the key factors involved are shown for *L*. *monocytogenes*, *S*. *flexneri*, *Rickettsia* spp., and *Burkholderia* spp. Host factors are indicated in red and bacterial factors are indicated in green. ERM, Ezrin, Radixin, Moesin Family Proteins; LLO, Listeriolysin O; Plc, phospholipase.

In conclusion and as shown in Fig 2, the host/pathogen interface supporting the first steps of bacterial dissemination, including cytosolic ABM, have been extensively investigated for *L*. *monocytogenes* and *S*. *flexneri* and are now fairly well understood for *Rickettsia* spp. and *Burkholderia* spp. By contrast, further investigation will be required to uncover the mechanisms supporting the formation and resolution of protrusions resolution and DMV escape for most pathogens that spread from cell to cell.
